# Increased interleukin-23 receptor^+ ^T cells in peripheral blood mononuclear cells of patients with systemic lupus erythematosus

**DOI:** 10.1186/ar3194

**Published:** 2010-11-29

**Authors:** Hathaipat Puwipirom, Nattiya Hirankarn, Pimpayao Sodsai, Yingyos Avihingsanon, Jongkonnee Wongpiyabovorn, Tanapat Palaga

**Affiliations:** 1 Lupus Research Unit, Chulalongkorn University, Phayathai Road, Pathumwan, Bangkok 10330, Thailand; 2 Medical Microbiology Interdisciplinary Program, Graduate School, Chulalongkorn University, Phayathai Road, Pathumwan, Bangkok 10330, Thailand; 3 Department of Microbiology, Faculty of Medicine, Chulalongkorn University, Phayathai Road, Pathumwan, Bangkok 10330, Thailand; 4 Department of Medicine, Faculty of Medicine, Chulalongkorn University, Phayathai Road, Pathumwan, Bangkok 10330, Thailand; 5 Department of Microbiology, Faculty of Science, Chulalongkorn University, Phayathai Road, Pathumwan, Bangkok 10330, Thailand

## Abstract

**Introduction:**

Systemic lupus erythematosus (SLE) is an autoimmune disorder characterized by production of autoantibodies and immune complex deposition in various organs. Aberrations in the T lymphocyte compartment and dysregulated cytokine production are key features of SLE pathogenesis and disease progression. Recently, the role of the interleukin (IL)-17/IL-23 axis in the pathogenesis of SLE has been reported. IL-23 and IL-23R are essential for expansion of pathogenic IL-17-producing T lymphocytes and have been shown to be important in the pathogenesis of lupus in animal models.

**Methods:**

In this study, the expression of IL-23R and IL-17 in CD4^+ ^and CD8^+ ^T lymphocytes in peripheral blood mononuclear cells (PBMCs) of SLE patients and control subjects were examined by flow cytometry. Twenty-nine SLE patients and 10 control subjects were recruited in this study. Patients were divided into active and inactive groups based on the SLE disease activity index (SLEDAI). As another disease control population, five psoriatic patients were recruited in this study.

**Results:**

Percentages of both IL23R^+ ^CD4^+ ^and IL-23R^+ ^CD8^+ ^T cell subsets were significantly higher in freshly isolated PBMCs from both groups of SLE patients compared to control subjects (*P *= 0.0021 and *P *= 0.0006, respectively). In addition, this difference was maintained after *ex vivo *stimulation with plate-bound anti-CD3/CD28 antibodies (*P *= 0.007 and *P *= 0.0019, respectively). When the fold increase in IL-17^+ ^T cells after *ex vivo *stimulation for three days was compared between patients and controls, SLE patients exhibited significantly higher increases in CD4^+ ^IL-17^+ ^and CD8^+ ^IL-17^+ ^T cells, suggesting that PBMCs from SLE patients promoted the expansion of IL-17-producing T cells upon stimulation more vigorously than control PBMCs. These trends were not observed in psoriasis patients. The correlations between IL-23R^+ ^T cells and IL-17^+ ^T cells and IL-23R^+ ^CD8^+ ^T cells and SLEDAI scores in patients were also found to be statistically significant.

**Conclusions:**

The results of our study confirmed the relevance of the IL-23/IL-17 axis in the pathogenesis of SLE and further highlighted the importance of IL-23R^+ ^T cell subsets in this autoimmune disease.

## Introduction

Systemic lupus erythematosus (SLE) is an autoimmune disorder that affects multiple organs and is characterized by production of autoantibodies and immune complex deposition in various organs, leading to inflammation and tissue destruction. T lymphocytes and their cytokines play essential roles in the immunopathogenesis of the disease [1]. Studies on cytokine profiles in SLE patients revealed a complex interplay between pro-inflammatory and anti-inflammatory cytokine networks [2]. It is, however, still controversial whether SLE can be simply categorized as a Th1/Th2 or other helper T cell type of autoimmune disease.

IL-23 is a heterodimeric cytokine produced predominantly by activated antigen presenting cells, such as macrophages and dendritic cells. The cytokine is composed of a unique p19 subunit and a p40 subunit that is shared with the Th1 signature cytokine, IL-12 [3]. Discovery of IL-23 led to the identification of a unique helper T cell subset called Th17 cells, which mainly produce the pro-inflammatory cytokines, IL-17 (A and F) and IL-21. IL-17 can be produced by several types of cells, including CD4^+ ^T cells (Th17), CD8^+ ^T cells, CD3^+ ^CD4^- ^CD8^- ^T cells, γ^δ ^T cells, NK cells and neutrophils [4]. IL-17 was initially associated with a Th1-type pro-inflammatory response and pathogenesis of Th1-type autoimmune diseases, but the realization that *IL-23p19- *and *IL-12p40-*deficient mice showed distinct phenotypes in terms of susceptibility to autoimmune diseases helped to establish Th17 as a unique helper T cell subset distinct from Th1 and Th2 cells [5,6]. The major transcription factors that regulate differentiation of Th17 cells are RORγt and RORα [7]. Cytokines produced by Th17 cells are highly pro-inflammatory and are now associated with various autoimmune diseases, such as psoriasis, rheumatoid arthritis and inflammatory bowel disease [4]. Differentiation of human naïve T cells into Th17 cells is regulated by TGFβ in the presence of IL-21 or the combinations of IL-6/IL-23/IL-1β and TNFα [8]. Although IL-23 plays only a minor role in the differentiation of Th17 from naïve T cells, it is necessary for driving the expansion of Th17 cells and is involved in the pathology of various autoimmune diseases [9]. Mechanisms leading to IL-17 production in other cell types besides helper T cells are not well understood.

The IL-23/IL-17 axis, therefore, is one of the main cytokine axes driving the pathogenesis of various autoimmune diseases, a role that had been previously attributed to Th1 cells. The receptor for IL-23 is composed of IL-12Rβ1, a common subunit shared with IL-12R, and a unique IL-23R [10]. IL-23R is not expressed in naïve T cells, but high levels of expression are found in activated/memory T cells. In addition to T cells, monocytes/macrophages, NK and dendritic cells also express IL-23R [11].

Since the identification of the Th17 lineage and its associated cytokines, multiple reports have documented the involvement of the IL-23/IL-17 axis in animal models of SLE and in SLE patients [4]. Sera IL-23 and IL-17 levels and the number of Th17 cells were elevated in SLE patients compared to control subjects [12]. Furthermore, the subset of CD4^- ^CD8^- ^double negative T cells was uniquely expanded and identified as a source of IL-17 in PBMCs and kidney biopsies from patients with SLE [13]. The results of most studies to date now imply that the cytokine milieu in SLE patients, such as low IL-2 and high IL-6/IL-21, may favor the differentiation of T cells into Th17 cells [2]. Taken together, the IL-23/IL-17 axis significantly contributes to the pathogenesis of SLE. In another autoimmune disease of chronic inflammatory skin, psoriasis, IL-23/Th17 axis is implicated in pathogenesis of the disease [14]. Increased mRNA expression of *IL-23p19 *and *IL-12p40 *were found in psoriatic skin lesions, and increased in circulating Th1, Th17 and Th22 were found in psoriasis [15]. In addition, single nucleotide polymorphisms in *IL23R *gene were reported to affect psoriatic phenotypes, which strongly link this disease with IL-23/Th17 axis [16].

In this study, we investigated the expression profile of IL-23R and IL-17 in T lymphocytes of PBMCs from SLE patients and examined its correlation with disease severity. We found significant increases in IL-23R^+ ^T lymphocytes in SLE patients regardless of disease severity, but this trend was not observed in psoriasis. Furthermore, we found significantly increased IL-17^+ ^CD4^+ ^and CD8^+ ^T cells in SLE patients and a positive correlations between IL-23R^+ ^and IL-17^+ ^T cells, IL-23R^+ ^T cells and SLEDAI scores in SLE patients.

## Materials and methods

### Patients and normal subjects

Twenty-nine Thai SLE patients were recruited from King Chulalongkorn Memorial Hospital and Bhumibol Adulyadej Hospital (Bangkok, Thailand). Diagnosis of SLE was established according to the Revised American College of Rheumatology (ACR) criteria, and disease activity was evaluated by the SLE disease activity index (SLEDAI) 2000 score. Active SLE disease was defined as a SLEDAI-2K score of ≥6 and inactive SLE disease was defined as a SLEDAI-2K score of <6 [17,18]. Recruited patients were classified into two groups, SLE patients with active SLE (active group, *n *= 13) and SLE patients with inactive SLE (inactive group, *n *= 16). Normal subjects were recruited as healthy controls. Psoriatic patients were recruited from King Chulalongkorn Memorial Hospital. The demographic data of subjects recruited in this study and the treatment each patient received are summarized in Table 1, 2 and 3. This study was approved by the Ethics Committee for Human Research of the Faculty of Medicine, Chulalongkorn University, and informed consent was obtained from all subjects.

**Table 1:** Demographic data of subjects recruited in this study

Characteristics	Active SLE Group	Inactive SLE Group	Psoriasis Group	Control Group
Numbers	13	16	5	10
Sex, female:male	12:1	16:0	3:2	10:0
Age, years	15 to 50	17 to 50	39 to 61	24 to 28
(mean ± S.D.)	(34.62 ± 11.46)	(33 ± 11.16)	(51 ± 9.88)	(25.6 ± 1.58)
SLEDAI score	≥ 6	< 6	-	-
(mean ± S.D.)	(12.92 ± 7.83)	(2.56 ± 2.03)		
Range of PASI score			11.2 to 27	
(mean ± S.D.)	-	-	(18.02 ± 5.38)	-
Treatment (mg/day)				
(mean ± S.D.)				
Prednisolone	13.13 ± 10.06	9.38 ± 8.45	-	-
Cyclophosphamide	-	25	-	-
Azathioprine	50 ± 30.62	47.5 ± 33.54	-	-
Mycophenolate mofetil	1,250	-	-	-
Methotrexate	-	-	6.43	

**Table 2:** Detailed characteristics of SLE patients included in this study

No. Patients	Stage	Treatments	Doses (mg/d)	SLEDAI score	Clinical Features
1	inactive	-	-	4	PI
2	inactive	PRD, AZT	5, 50	0	-
3	inactive	PRD, AZT	35, 12.5	4	RA, MU
4	inactive	PRD, AZT	5, 25	1	FE
5	inactive	PRD	N/A	5	RA, MU, FE
6	inactive	PRD	2.5	0	-
7	inactive	PRD, CPM	10, N/A	4	PU
8	inactive	PRD	5	4	AR
9	inactive	-	-	4	PU
10	inactive	PRD	5	0	-
11	inactive	PRD, AZT	10, 50	4	PU
12	inactive	PRD, CPM	7.5, 25	0	-
13	inactive	PRD, CPM	10, 25	4	PU
14	inactive	PRD	10	2	MU
15	inactive	PRD, AZT	7.5, 100	5	PU, LP
16	inactive	-	-	0	-
17	active	PRD, AZT	5, 100	8	HE, PU
18	active	-	-	8	HE, PU
19	active	PRD, MMF	2.5, 2,000	8	HE, PU
20	active	PRD	15	30	SZ, UC, HE, PU, PI, PE, LC, IDB
21	active	PRD, AZT	5, 25	11	MY, HE, PL, LP
22	active	PRD	5	8	AR, PU
23	active	PRD, AZT	30, 50	29	VA, AR, HE, PU, PI, RA, MU, LP
24	active	PRD	30	11	PU, AL, PL, FE, TC, LP
25	active	PRD, AZT	5, 50	8	VA
26	active	PRD, MMF	5, 500	13	HE, PU, AL, MU, LP
27	active	PRD	20	12	HE, PU, PI
28	active	PRD, AZT	20, 25	16	VA, PU, MU, LC
29	active	PRD	15	6	PU, LC

AL, alopecia; AR, arthritis; AZT, azathioprine; CPM, cyclophosphamide; FE, fever; HE, hematuria; IDB, Increased DNA binding; LC, low complement; LP, leucopenia; MMF, mycophenolate mofetil; MU, mucosal ulcers; MY, myositis; N/A, data not available; PE, pericarditis; PI, pyuria; PL, pleurisy; PRD, prednisolone; PU, proteinuria; RA, rash; SZ, seizure; TC, thrombocytopenia; UC, urinary casts; VA, vasculitis.

**Table 3:** Detailed characteristics of psoriasis patients included in this study

No. of Patients	PASI Score	Treatments	Doses (mg/d)
1	20.5	No treatment	-
2	15.2	Methotrexate	2.14
3	11.2	No treatment	-
4	16.2	Methotrexate	10.71
5	27	Photo NB-UVB	-

### Antibodies

For cell surface staining, anti-CD4-APC and anti-CD8-PerCP antibodies and FITC conjugated-streptavidin were purchased from BD Biosciences (San Diego, CA, USA); biotinylated anti-IL-23 receptor antibody was obtained from R&D Systems (Minneapolis, MN, USA). Staining for intracellular cytokines was performed with the anti-IL-17A-PE antibody purchased from eBioscience (San Diego, CA, USA).

### Isolation and *ex vivo *culture of PBMCs

Seventeen milliliters of venous peripheral blood from patients and normal subjects was collected in ACD blood collection tubes (BD Pharmingen, San Diego, CA, USA). PBMCs were isolated by Ficoll-Hypaque Isoprep (Robbins Scientific, Sunnyvale, CA, USA). Isolated PBMCs (1 × 10^6 ^cells/ml) were immediately stained with cell surface markers and intracellular cytokines (referred to as Day 0) or incubated (1 × 10^6 ^cells/ml/well) in 24-well tissue culture plates pre-coated with anti-CD3 and anti-CD28 antibodies (1 μg/ml) (BD Pharmingen) in RPMI-1640 medium supplemented with 10% heat-inactivated fetal bovine serum, 100 U/mL of penicillin, 100 μg/ml of streptomycin and non-essential amino acids (GIBCO BRL, Karlsruhe, Germany) for 72 hours (referred to as Day 3). *Ex vivo *stimulated PBMCs were harvested and stained with cell surface markers and intracellular cytokines using the antibodies described above, and the data were analyzed by flow cytometry.

### Cell surface and intracellular cytokine staining

Cells treated as indicated were resuspended in staining buffer (PBS supplemented with 0.02% sodium azide and 1% BSA). The conjugated antibodies for cell surface staining were added and cells were incubated at 4°C in the dark for 30 minutes. This step was followed by washing and fixing in 4% paraformaldehyde (Sigma-Aldrich, St. Louis, MO, USA) for 20 minutes at room temperature in the dark. Fixed cells were washed and resuspended in permeabilization buffer (PBS supplemented with 0.04% sodium azide, 0.1% BSA and 0.1% saponin) for 10 minutes at room temperature in the dark. The anti-IL-17-PE antibody was added to cells and the mixture was incubated for 30 minutes at 4°C in the dark before analysis on a flow cytometer. All samples were analyzed on a four-color FACSCalibur (BD Biosciences). Data analysis was performed with Summit software 5.0 (Dako, Glostrup, Denmark).

### Statistical analysis

Statistical analysis was performed using GraphPad Prism 4 software (GraphPad Software, San Diego, CA, USA). Differences among groups were compared using the Mann-Whitney U-test. Spearman's rank correlation test was used to assess the correlation of two variables. Probability values of *P *< 0.05 were considered statistically significant. All probabilities were two-tailed.

## Results

### Increased IL-23R^+ ^T lymphocytes in PBMCs from SLE patients

**Figure 1:**
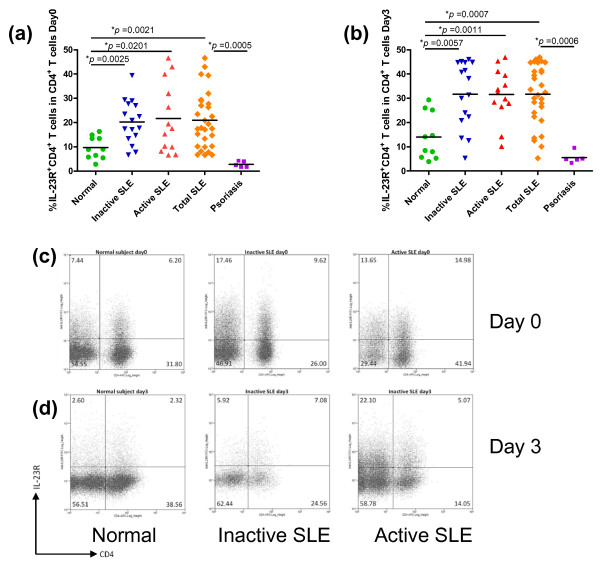
**Increased percentages of IL-23R^+ ^CD4^+ ^T cells in PBMCs from SLE patients**. **(a, b) **The percentages of IL-23R^+ ^CD4^+ ^T cells in PBMCs from inactive and active SLE patients, psoriatic patients and normal subjects on Day 0 (a) and Day 3 (b) are shown. The horizontal bars show the mean values. The percentages of IL-23R^+ ^CD4^+ ^T cells were calculated from total CD4^+ ^T cells after lymphocyte gating. **(c, d) **Representative flow cytometric profiles of IL-23R^+ ^CD4^+ ^T cells in PBMCs from inactive (Day 0: middle panel in (c), Day 3: middle panel in (d)) and active (Day 0: right panel in (c), Day 3: right panel in (d)) SLE patients and normal subjects (Day 0: left panel in (c), Day 3: left panel in (d)) are shown.

**Figure 2:**
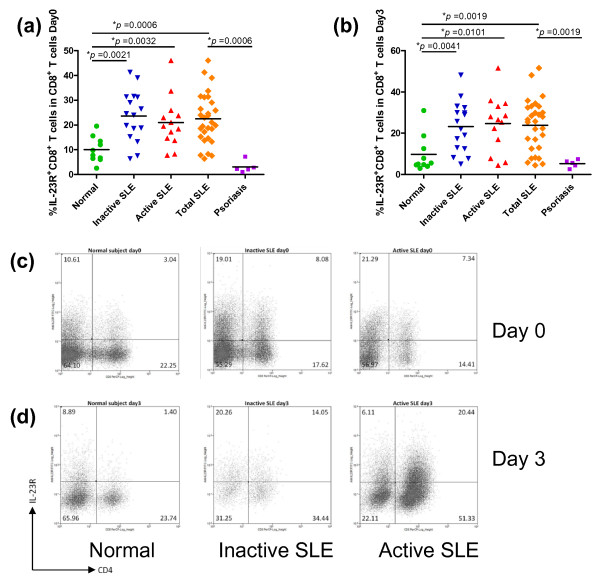
**Increased percentages of IL-23R^+ ^CD8^+ ^T cells in PBMCs from SLE patients**. **(a, b) **The percentages of IL-23R^+ ^CD8^+ ^T cells in PBMCs from inactive and active SLE patients, psoriatic patients and normal subjects on Day 0 (a) and Day 3 (b) are shown. The horizontal bars show the mean values. The percentages of IL-23R^+ ^CD8^+ ^T cells were calculated from total CD8^+ ^T cells after lymphocyte gating. **(c, d) **Representative flow cytometric profiles of IL-23R^+ ^CD8^+ ^T cells in PBMCs from inactive (Day 0: middle panel in (c), Day 3: middle panel in (d)) and active (Day 0: right panel in (c), Day 3: right panel in (d)) SLE patients and normal subjects (Day 0: left panel in (c), Day 3: left panel in (d)) are shown.

To investigate the expression profile of IL-23R and IL-17A (referred to as IL-17) in T lymphocytes from SLE patients, PBMCs were isolated and immediately stained for cell surface markers (CD4, CD8 and IL-23R) and intracellular cytokines (IL-17) (Day 0). The percentages of CD4^+ ^IL-23R^+ ^and CD8^+ ^IL-23R^+ ^T lymphocytes were analyzed after gating on lymphocytes. As shown in Figures 1a, c and 2a, c, PBMCs from all SLE patients, regardless of disease activity based on SLEDAI score, exhibited significantly higher percentages of cells that were CD4^+ ^IL-23R^+ ^and CD8^+ ^IL-23R^+ ^than control subjects (*P *= 0.0021 for CD4^+ ^and *P *= 0.0006 for CD8^+ ^T cells). When SLE patients were divided into active and inactive groups based on SLEDAI scores, differences between each patient group and the control subjects remained statistically significant (Figures 1a and 2a). However, differences between SLE patients in the active disease stage and inactive disease stage were not detected.

Because all T cells from PBMCs may not be in a stage of activation, we wondered whether the differences observed above between SLE patients and normal controls would be more evident after polyclonal activation *ex vivo*. Upon *ex vivo *stimulation by plate-bound anti-CD3 and anti-CD28 antibodies for three days (Day 3), the higher proportion of CD4^+ ^IL-23R^+ ^and CD8^+ ^IL-23R^+ ^in all SLE patients remained statistically significant compared to the control group (Figure 1b, d and Figure 2b, d; *P *= 0.0007 and *P *= 0.0019, respectively). Similar to the results obtained from Day 0, no statistical difference was found between inactive and active patients in percentages of IL-23R^+ ^cells at Day 3. This may be due to the effect of the higher dose of immunosuppressive drugs given to patients in the active group. When we compared data from SLE patients with those from psoriatic patients, psoriatic patients showed significantly lower percentages of IL-23R^+ ^T cells than SLE patients (Figures 1a, b and 2a, b). The results described here strongly suggest that SLE patients have a higher frequency of IL-23R^+ ^T lymphocytes in PBMCs, both in CD4^+ ^and CD8^+ ^subsets, than the control group and psoriatic group, and this difference was maintained after *ex vivo *stimulation.

### Increase in IL-17^+ ^T lymphocytes in SLE patients upon *ex vivo *activation

**Figure 3:**
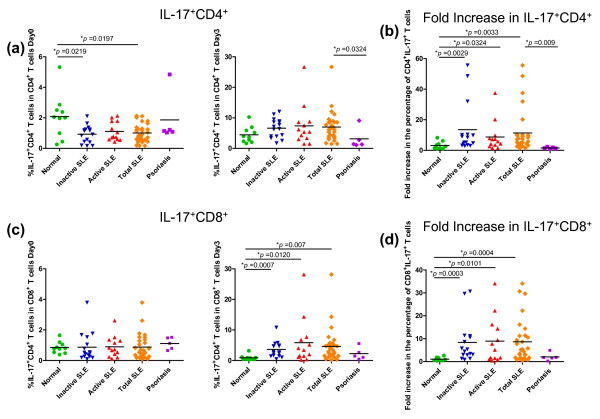
**Increased percentages of IL-17^+ ^CD4^+ ^T cells and IL-17^+ ^CD8^+ ^T cells in PBMCs from SLE patients**. **(a, c) **The percentages of IL-17^+ ^CD4^+ ^T cells (a) or IL-17^+ ^CD8^+ ^T cells (c) in PBMCs from inactive and active SLE patients, psoriatic patients and normal subjects on Day 0 (left panel) and Day 3 (right panel) are shown. The horizontal bars show the mean values. **(b, d) **The fold increase in the percentages of IL-17^+ ^CD4^+ ^T cells (b) and IL-17^+ ^CD8^+ ^T cells (d) in PBMCs from inactive and active SLE patients, psoriatic patients and normal subjects is shown. The percentages of IL-17^+ ^T cells at Day 3 were divided by those of Day 0 from individual samples and the results were presented as fold increase. The horizontal bars show the mean values.

Previous reports documented an increase in IL-17^+ ^T lymphocytes in PBMCs and kidney infiltrates in SLE patients [13]. When IL-17^+ ^T cells were assessed in the same manner as described above, percentages of IL-17^+ ^CD4^+ ^and IL-17^+ ^CD8^+ ^T cells freshly isolated were not different between control subjects and SLE patients (Figure 3a, c). In fact, some control subjects had higher percentages of IL-17^+ ^CD4^+ ^T cells than SLE patients. At Day 3, after *ex vivo *stimulation, PBMCs from SLE patients showed significantly higher percentages of IL-17^+ ^CD8^+ ^T cells than controls (*P *= 0.0007) but no difference from controls in percentages of CD4^+ ^IL-17^+ ^cells (Figure 3b).

More importantly, however, when the fold increase in CD4^+ ^IL-17^+ ^and CD8^+ ^IL-17^+ ^subsets from Day 0 to Day 3 was analyzed, it was found that both CD4^+ ^and CD8^+ ^subsets had significantly higher IL-17^+ ^staining than the control subjects (Figure 3c, d; *P *= 0.0033 for CD4^+ ^and *P *= 0.0004 for CD8^+^). Because the difference in IL-17^+ ^T cell subsets between patients and the control group was not found at Day 0, the higher percentages of IL-17^+ ^T cells after *ex vivo *stimulation strongly implies that T cells in the PBMCs of SLE patients preferentially skew towards IL-17 production and expand more vigorously than those of the control, both in the CD4^+ ^and CD8^+ ^compartments. Investigation into whether all IL-17^+ ^T cells were limited to the IL-23R^+ ^subset revealed that IL-17^+ ^T cells exhibited both an IL-23R^+ ^and IL-23R^- ^phenotype (data not shown). No differences were found between normal group and psoriatic patient group (Figure 3), suggesting that the characteristic of fold increase in IL-17^+ ^T cells upon *ex vivo *stimulation is specific for SLE but not psoriasis.

### Correlation between percentages of IL-23R^+ ^T cells and IL-17^+ ^T cells in SLE patients

**Figure 4:**
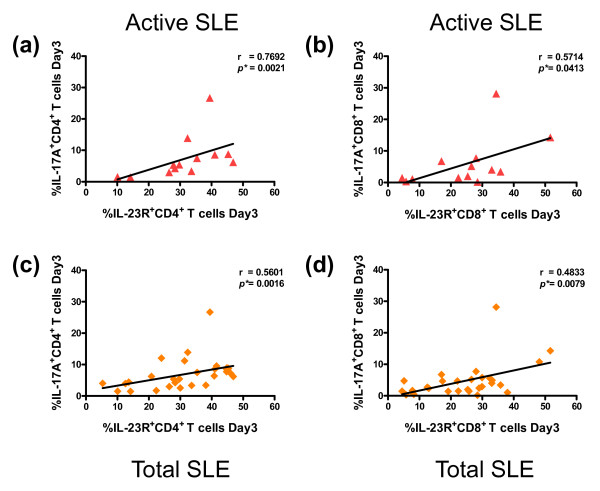
**Correlation between percentage of IL-23R^+ ^T cells and percentage of IL-17^+ ^T cells in SLE patients**. **(a, c) **Relationships between the percentage of IL-23R^+ ^CD4^+ ^T cells and the percentage of IL-17^+ ^CD4^+ ^T cells on day 3 in active (a) and total SLE (c) groups (r = 0.7692, *P *= 0.0021 and r = 0.5601, *P *= 0.0016, respectively) are shown. **(b, d) **A relationship between the percentage of IL-23R^+ ^CD8^+ ^T cells and the percentage of IL-17^+ ^CD8^+ ^T cells on Day 3 was also found in active (b) and total SLE (d) groups (r = 0.5714, *P *= 0.0413 and r = 0.4833, *P *= 0.0079, respectively) are shown. Spearman's correlation test was used to analyze these data.

**Figure 5:**
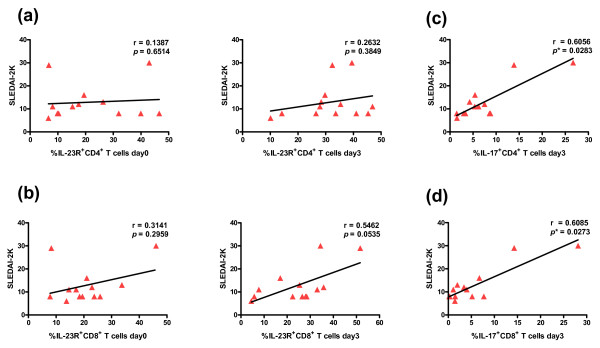
**Correlation between percentages of IL-23R^+ ^T cells and SLEDAI and IL-17^+ ^T cells and SLEDAI in active SLE patients**. **(a, b) **Relationships between the percentages of IL-23R^+ ^CD4^+ ^or CD8^+ ^T cells on Day 0 and Day 3 and SLEDAI in active SLE groups are shown. **(c, d) **Relationships between the percentages of IL-17^+ ^T CD4^+ ^or CD8^+ ^T cells on Day 3 from SLE patients (r = 0.6056, *P *= 0.0283 for CD4^+ ^and r = 0.6085, *P *= 0.0273 for CD8^+^) are shown.

When the correlation between percentages of IL-23R^+ ^T cells and IL-17^+ ^T cells was analyzed, a significant and positive correlation was found in percentages of CD4^+^IL-23R^+ ^T cells and CD4^+^IL-17^+ ^T cells at Day 3 in the active SLE group (r = 0.7692, *P *= 0.0021) and in total SLE cases (r = 0.5601, *P *= 0.0016) (Figure 4a, c). Similarly, a significant and positive correlation was also found in CD8^+ ^T cells at Day 3 in active and total SLE patients (r = 0.5174, *P *= 0.0413 for active SLE and r = 0.4833, *P *= 0.0079 for total SLE) (Figure 4b, d). The correlation of IL-23R^+ ^T cells and IL-17^+ ^T cells in the inactive group and control subjects did not reach statistical significance (data not shown). In addition, when we analyzed the correlation between disease severity using SLEDAI score and percentages of IL-23R^+ ^T cells or IL-17^+ ^T cells in SLE patients, only the positive correlation between SLEDAI and IL-23R^+^CD8^+ ^T cells on Day 3 in active SLE group was found (r = 0.5462, *P *= 0.0535), but it did not reached statistical significance (Figure 5b). Furthermore, the correlation between percentages of IL-17^+ ^T cells and SLEDAI scores were observed only in active SLE group (r = 0.6056, *P *= 0.0283 for CD4^+ ^and r = 0.6085, *P *= 0.0273 for CD8^+^) (Figure 5c, d). These results strongly suggest that higher IL-23R^+ ^T cells and higher IL-17^+ ^T cells (both CD4^+ ^and CD8^+ ^subsets) are one of the characteristics exhibited by SLE patients and may be useful biomarkers for detection of severity and disease stage in SLE patients.

## Discussion

Even though the protective role of the IL-23/IL-17 axis is essential for certain bacterial and fungal infections, its contribution to the immunopathology of various autoimmune diseases has been highlighted [19]. Recent evidence, both in lupus animal models and human SLE, strongly links the pathogenesis of SLE with the IL-23/IL-17 axis [20]. Most studies have found higher numbers of Th17 cells or elevated Th17 cytokines in SLE patients or lupus prone mice [12,13]. Th17-derived cytokines not only trigger vicious inflammatory cycles but also seem to provide help for B cells, which leads to production of autoantibodies [2].

In this study, we found that PBMCs from SLE patients contained higher percentages of IL-23R^+ ^T lymphocytes as compared to those of the control subjects. Expression of IL-23R is reported to be restricted to effector and memory T lymphocytes and possibly to Th17 lineage cells. Therefore, our data suggest that SLE patients may have high numbers of effector and memory Th17 cells in circulation. This is consistent with a previous study using a lupus animal model, which showed that IL-23R-deficiency prevented the development of lupus nephritis in *lpr*/*lpr *mice [21]. Upon *ex vivo *stimulation, the percentages of IL-23R^+ ^T lymphocytes in SLE patients remained higher than those of the control subjects. Because IL-23R can be expressed in both effector and memory cells, it will be of interest to test whether subsets of cells we detected in this study have effector or memory phenotypes. Human Th17 cells selectively expressing IL-17 with a memory phenotype can be further identified by chemokine markers, including CCR6 and CCR4 [22].

When IL-17 was assessed in samples of freshly isolated PBMCs, we did not detect any statistical significance between controls and patients. Similarly, we found no detectable level of IL-17 in the sera of patients in our study; when sera IL-17 was measured by ELISA, only two sera samples from SLE patients yielded detectable IL-17 (5.12 and 6.17 pg/ml), while the rest of the samples from both the SLE patients and control subjects did not reach detectable levels (data not shown). In contrast, other studies have reported the elevation of both IL-17 and IL-23 in sera from patients [12]. One report, however, observed similar results in SLE patients in which no difference in sera IL-17 was found [23]. The reasons for the discrepancy between our results and others are not known but may be due to the effects of drug regimens patients recruited in this study received. Interestingly, when we compared the fold increase in IL-17^+ ^T cells from Day 0 to Day 3 upon *ex vivo *PBMCs stimulation, significant differences were detected between the control group and SLE patients. The positive correlation between IL-23R^+ ^and IL-17^+ ^T cells in SLE patients further highlighted the importance of IL-23R^+ ^IL-17^+ ^T cells in this disease. These data suggest that IL-17^+ ^T cells vigorously expanded during *ex vivo *stimulation and probably reflect the higher frequency of IL-23R^+ ^T cells in freshly isolated PBMCs from SLE patients. When we analyzed data from psoriatic patients, no detectable increase in either IL-23R^+ ^or IL-17^+ ^T cells were found. Since psoriasis is an autoimmune disease with both genetic and immunopathogenic links with IL-23/Th17 axis, this is somewhat unexpected. However, most reports on the links between psoriasis and increases in cells or molecules of the IL-23/Th17 axis focused on psoriatic skin lesions, and the report on increase in circulating Th17 in psoriasis did not use IL-23R but other markers to identify the Th17 population [15,24]. Therefore, it is possible that increased frequency in IL-23R^+ ^T cells may be a specific characteristic of SLE but not psoriasis.

When SLE patients recruited in this study were classified into active and inactive groups based on disease severity by SLEDAI score, the correlation between disease severity and percentages of IL-23R^+ ^or IL-17^+ ^T cells was not found in inactive SLE groups, but the correlation were found in active SLE patients between IL-23R^+^CD8^+ ^T cells only after *ex vivo *stimulation. Even though this correlation did not reach statistical significance, this may imply that higher percentages of IL-23R^+ ^T lymphocytes *per se *may not directly promote pathogenesis, but perhaps the cytokines produced by these cells act as effector molecules in the pathogenesis of SLE. Upon *ex vivo *stimulation, these pathogenic groups of cells are probably selectively expanded. Therefore, as long as the immunosuppressive drugs keep the cytokine-producing effector cells in check, the disease outcome may not be as severe, but the subsets of cells ready to produce such pro-inflammatory cytokines may be present in patients even when the disease is in an inactive stage.

In addition, we also found that CD8^+ ^T lymphocytes with an IL-23R^+ ^phenotype were higher in SLE patients. Whether this subset of CD8^+ ^T cell also produces IL-17 *in vivo *and whether it plays a role in the pathogenesis of SLE have not been documented. Recent evidence suggests that IL-23/IL-23R drives pathogenic IL-17-producing CD8^+ ^T cells [25]. Fold increases in CD8^+ ^T cells positive for IL-17 were higher in SLE patients. Interestingly, expanded, double-negative αβ T cells in SLE patients have been found to produce IL-17 and infiltrate the kidneys, and this subset of cells can be derived from CD8^+ ^T cells with an inflammatory effector phenotype [13,26]. From this point of view, the result from our study is consistent with these reports, and increased IL-23R^+ ^CD8^+ ^T cells may become double-negative T cells upon stimulation.

Taken together, we added another supportive piece of evidence for the role of the IL-23/IL-17 axis in SLE, suggesting that these cytokines may play a central role in the pathogenesis of the disease. The results from our study may be useful for therapeutic intervention in SLE patients.

## Conclusions

SLE patients, regardless of disease severity, have higher percentages of IL-23R^+ ^T lymphocytes (both CD4^+ ^and CD8^+ ^subsets) than the control subjects in PBMCs and psoriatic patients. Upon *ex vivo *stimulation, PMBCs from SLE patients preferentially expanded IL-17 producing T cells, when compared with the normal subjects. The statistically significant correlation between percentages of IL-23R^+ ^T cells and IL-17^+ ^T cells, and IL-17^+ ^T cells and SLEDAI scores were found in active SLE patients. Taken together, this study suggests that the IL-23/IL-17 axis is implicated in the pathogenesis of SLE.

## Abbreviations

IL: interleukin; PMBCs: peripheral blood mononuclear cells; SLE: systemic lupus erythematosus; SLEDAI: SLE disease activity index.

## Competing interests

The authors declare that they have no competing interests.

## Authors' contributions

All authors contributed to the design of the study, and to the acquisition and interpretation of data. HP and PS carried out the analysis by flow cytometry. YA, NH and JW evaluated patients' data. TP and NH drafted the manuscript. All authors read and approved the final manuscript.
